# Sperm Proteasomes Degrade Sperm Receptor on the Egg Zona Pellucida during Mammalian Fertilization

**DOI:** 10.1371/journal.pone.0017256

**Published:** 2011-02-23

**Authors:** Shawn W. Zimmerman, Gaurishankar Manandhar, Young-Joo Yi, Satish K. Gupta, Miriam Sutovsky, John F. Odhiambo, Michael D. Powell, David J. Miller, Peter Sutovsky

**Affiliations:** 1 Division of Animal Science, and Departments of Obstetrics, Gynecology, and Women's Health, University of Missouri–Columbia, Columbia, Missouri, United States of America; 2 Research Center for Transgenic Cloned Pigs, Chungnam National University, Daejeon, South Korea; 3 Reproductive Cell Biology Laboratory, National Institute of Immunology, Aruna Asaf Ali Marg, New Delhi, India; 4 Morehouse School of Medicine, Morehouse Univeristy, Atlanta, Georgia, United States of America; 5 Department of Animal Sciences, University of Illinois at Urbana–Champaign, Urbana, Illinois, United States of America; University of South Florida College of Medicine, United States of America

## Abstract

Despite decades of research, the mechanism by which the fertilizing spermatozoon penetrates the mammalian vitelline membrane, the zona pellucida (ZP) remains one of the unexplained fundamental events of human/mammalian development. Evidence has been accumulating in support of the 26S proteasome as a candidate for echinoderm, ascidian and mammalian egg coat lysin. Monitoring ZP protein degradation by sperm during fertilization is nearly impossible because those few spermatozoa that penetrate the ZP leave behind a virtually untraceable residue of degraded proteins. We have overcome this hurdle by designing an experimentally consistent *in vitro* system in which live boar spermatozoa are co-incubated with ZP-proteins (ZPP) solubilized from porcine oocytes. Using this assay, mimicking sperm-egg interactions, we demonstrate that the sperm-borne proteasomes can degrade the sperm receptor protein ZPC. Upon coincubation with motile spermatozoa, the solubilized ZPP, which appear to be ubiquitinated, adhered to sperm acrosomal caps and induced acrosomal exocytosis/formation of the acrosomal shroud. The degradation of the sperm receptor protein ZPC was assessed by Western blotting band-densitometry and proteomics. A nearly identical pattern of sperm receptor degradation, evident already within the first 5 min of coincubation, was observed when the spermatozoa were replaced with the isolated, enzymatically active, sperm-derived proteasomes. ZPC degradation was blocked by proteasomal inhibitors and accelerated by ubiquitin-aldehyde(UBAL), a modified ubiquitin protein that stimulates proteasomal proteolysis. Such a degradation pattern of ZPC is consistent with in vitro fertilization studies, in which proteasomal inhibitors completely blocked fertilization, and UBAL increased fertilization and polyspermy rates. Preincubation of intact zona-enclosed ova with isolated active sperm proteasomes caused digestion, abrasions and loosening of the exposed zonae, and significantly reduced the fertilization/polyspermy rates after IVF, accompanied by en-mass detachment of zona bound sperm. Thus, the sperm borne 26S proteasome is a candidate zona lysin in mammals. This new paradigm has implications for contraception and assisted reproductive technologies in humans, as well as animals.

## Introduction

Mammalian spermatozoa become fertilization-competent during capacitation in the female oviduct, a process that alters the sperm motility pattern and primes the sperm exocytotic organelle, the sperm head acrosome, for interactions with the egg zona pellucida (ZP)[1], [2]. Upon binding to the sperm receptor on the mammalian ZP (ZPC protein in the mouse or ZPB-ZPC heterodimer in the pig), the fertilizing spermatozoon undergoes acrosomal membrane vesiculation and exocytosis of the acrosomal cap, referred to as the acrosome reaction or acrosomal exocytosis (AE)[3], [4]. This event results in the formation of the acrosomal shroud, a sperm head-enveloping cluster of acrosomal membrane vesicles and matrices that exposes the acrosome-borne proteolytic enzymes. The AE enables the spermatozoon to proceed with the formation of a fertilization slit and the penetration of the ZP [3]. Despite four decades of intense research, the mechanism of mammalian sperm-zona penetration remains elusive [5], [6]. Currently two different schools of thought interpret this major unresolved issue in developmental biology: Proponents of mechanical penetration hold that the motile force exerted from the sperm tail is sufficient to push the fertilizing spermatozoon through the ZP [7], [8]. However, physical forces generated by the sperm flagellum do not appear to fully account for sperm ability to push through the thick ZP [9]. The second theory, introduced as early as 1958 by Austin and Bishop [10], proposes that the fertilizing spermatozoa release an enzyme, a putative zona “lysin”, present in the sperm head acrosomal matrix [10], [11].

While the acrosomal protease acrosin was initially ruled out as a crucial enzyme in such a scheme [12], [13], the 26S proteasome has been gaining favor as a candidate mammalian, ascidian and invertebrate vitelline membrane lysin (reviewed in [14], [15]). It is hypothesized that the sperm acrosome-borne proteasomes degrade a sperm receptor protein on the ZP that becomes ubiquitinated either during oogenesis (as in echinoderms and mammals) [16], [17] or directly by the sperm-released ubiquitination machinery during fertilization (as in ascidians)[18]. Recently, this hypothesis has been supported by yeast two-hybrid studies in which a proteasome-interacting, ubiquitin-binding protein UBAP2L has been identified as the most likely ZPC-interacting protein in human spermatozoa [19].

**Figure 1:**
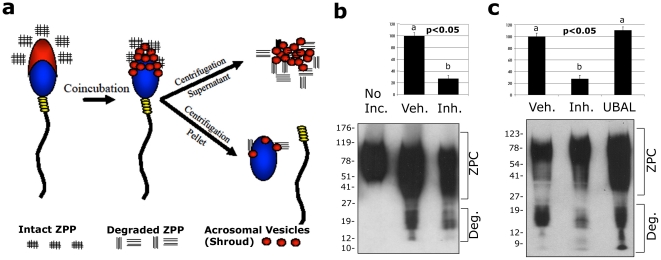
Acrosome-borne 26S proteasomes degrade sperm receptor protein ZPC, solubilized from mature oocytes' zonae. (**a**) Diagram of the experimental setup in which the ZP-proteins (ZPP), solubilized by non-degrading/non-reducing methods from 100 meiotically mature, fertilization-competent porcine oocytes, were coincubated for 2 h with 10,000 capacitated boar spermatozoa. Each lane in the Western blots shown in panels b & c represent this number of gametes/proteins. The ZPP binding induced acrosomal exocytosis, enabling the separation of ZPC and sperm proteasome-containing acrosomal shrouds from the exocytosed spermatozoa. (**b**) Western blotting of ZPC in these co-incubated fractions detected a low mass degradation product (“Deg.” bracket), which was present in the coincubated fraction after 2 h (“Veh.” lane) or after 2 h of coincubation in the presence of a proteasomal inhibitor cocktail composed of epoxomicin, clasto-lactacystin-beta lactone (CLβL) and MG132 (“Inh.” lane), but not detectable in the ZPP fraction prior to coincubation (“No Inc.” lane). Densitometry revealed a statistically significant reduction (73% over the vehicle lane) in the density of the measured ZPC-degradation product (data from three replicates). (**c**) Accelerated degradation of ZPC was accomplished with the addition of ubiquitin-aldehyde (UBAL), a modified ubiquitin molecule that increases proteasomal proteolytic activity. Note a unique, low molecular mass band of <9 kDa that is prominent in the UBAL lane.

Historically, it has been difficult to prove that mammalian ZP proteins and those with sperm-receptor function in particular, are degraded by the enzymes originating from the sperm acrosome during fertilization. Only one or very few spermatozoa actually penetrate ZP during fertilization and leave a virtually untraceable residue of degraded ZP proteins. We have overcome this hurdle by designing an experimentally consistent *in vitro* system (**Fig. 1a**) in which 10,000 live, freshly collected (never cryospreserved) and capacitated (i.e. fertilization competent) boar spermatozoa are co-incubated with ZP-proteins (ZPP) solubilized by non-degrading/non-reducing methods from 100 meiotically mature, fertilization-competent porcine oocytes. Upon co-incubation, the soluble ZP-proteins bind to sperm acrosomal surface receptors as they would during fertilization and induce the process of acrosomal exocytosis, including the fusion of plasma and outer acrosomal membranes that results in the formation of the membrane vesicle-composed acrosomal shroud. This enables the interaction of acrosomal enzymes with ZPP. In the present study, this fertilization relevant *in vitro* assay is used to demonstrate the ability of sperm-borne proteasomes to degrade the mammalian sperm receptor on the oocyte zona pellucida.

## Results

### Sperm-Borne Proteasomes Degrade Solubilized Zona Proteins *In Vitro*

Western blot analysis of porcine sperm receptor component ZPC was conducted on the supernatant fraction which, after coincubation, contained partially degraded ZP proteins as well as acrosomal shrouds of acrosome-reacted spermatozoa. We submitted these co-incubated fractions to SDS-PAGE and Western blotting densitometry with a peptide-specific anti-ZPC antibody recognizing the N-terminal domain (QPVWQDEGQRLR sequence; aa 23-34) of secreted porcine ZPC, and known to recognize proteolytic fragments of ZPC [20], [21], [22](see **Fig. S1** for zona staining with this antibody). We were thus able to trace the ZP degradation by spermatozoa (**Fig. 1b, c**) on a large scale (compared to fertilization). The proteolysis of ZPC was significantly inhibited in the presence of a cocktail of three specific proteasomal inhibitors, epoxomicin, clasto-lactacystin-beta lactone (CLβL) and MG132 (**Fig. 1b**). In turn, we accelerated the degradation of ZPC with ubiquitin-aldehyde (UBAL), a modified ubiquitin molecule that inhibits the proteasome-associated deubiquitinating enzymes, but increases proteasomal proteolytic activity (**Fig. 1c**). Comparison of the vehicle control group to the proteasomal inhibitor-treated group revealed a significant difference (p<0.05) in the density of ZPC-degradation product within the 12–19 kDa range (**Fig. 1b**). Proteasomal inhibitors reduced the degradation product by 73% (**Fig. 1b**). Further analysis of ZPC degradation revealed a unique, low molecular mass band of <9 kDa, present only in the UBAL-treated group (**Fig. 1c**).

### Proteasomal Inhibitors Prevent the Breakdown of Acrosomal Shrouds Induced by Sperm Coincubation with Solubilized Zonae

**Figure 2:**
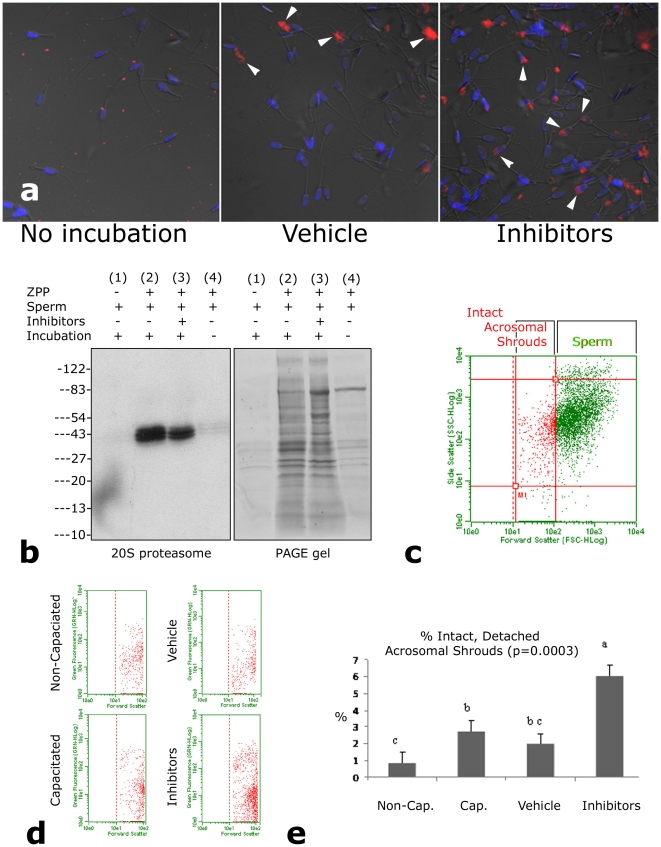
Soluble ZPC protein binds to capacitated spermatozoa and triggers acrosomal exocytosis and separation of the acrosomal shrouds. (**a**). Immunofluorescence of sperm receptor protein ZPC (red) immediately after ZPP-sperm mixing (left), after 2 h of incubation (center; note the detached acrosomal shrouds-arrowheads) and after 2 h incubation with proteasomal inhibitors (right; arrows point to sperm with attached shrouds). (**b**). Addition of proteasomal inhibitors to sperm-ZPP coincubation limited the release of acrosomal proteasomes. Supernatant fractions (left panel) were collected from 10,000 sperm prior to mixing with ZPP (lane 1), after 2 h of coincubation (lane 2; appropriate vehicles were present), after 2 h co-incubation with proteasomal inhibitors added (lane 3) and immediately after sperm-ZPP mixing. Proteasomes were detected with a monoclonal antibody against alpha-type 20S proteasomal core subunits. The right panel shows the corresponding residual PAGE gel after protein transfer, confirming comparable protein loads between vehicle and inhibitor lanes (lanes 2&3). Lanes 1 and 4 contain only a small amount of protein because of limited acrosomal exocytosis. (**c-e**) Sperm acrosomal status and the formation and detachment of acrosomal shrouds upon co-incubation were monitored by flow cytometry of live spermatozoa in which the acrosomes were labeled with fluorescently-conjugated lectin PNA. (**c**) Gating of detached acrosomal shrouds (red dots) from spermatozoa (green dots) in scatter diagrams from flow cytometry of sperm-ZPP fractions. Each dot represents one flow cytometric event, a shroud or a sperm cell (2,000 events/fraction). (**d**) Number of intact acrosomal shrouds, gated in visible light scatter, is increased by addition of proteasomal inhibitors (lower right panel) to coincubation, compared to control sperm-ZPP fraction (upper right), and sperm fractions prior to (upper left) and after capacitation (upper right), not exposed to ZPP. A degree of spontaneous acrosomal exocytosis is expected during capacitation. (**e**) Percentage of detached acrosomal shrouds in the coincubation fraction (i.e. ratio of red dot-events to all events in the scatter diagrams gated on acrosomal shrouds), was increased significantly (ANOVA; p<0.05) by the addition of proteasomal inhibitor cocktail for the duration of sperm-ZPP coincubation.

**Figure 3:**
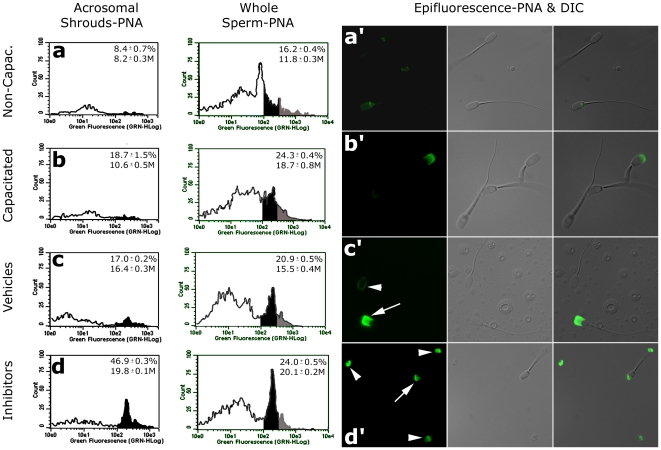
The ZPP-induced formation, detachment and breakdown of acrosomal shrouds traced by flow cytometry and epifluorescence microscopy with lectin PNA-FITC. Lectin PNA binds to terminal β-galactose residues of disaccharides present on the outer acrosomal membrane (OAM), which is concealed in the non-capacitated spermatozoa, partially exposed in the capacitated ones and completely exposed during acrosomal exocytosis, which encompasses the vesiculation of OAM, and formation and eventual breakdown of the acrosomal shroud. During fertilization, the shroud remains attached to egg coat surface, forming a viscous cloud around the penetrating sperm head [1]. In a cell free system with live sperm and soluble ZPP, the shrouds eventually detach from the sperm heads of capacitated, ZPP-exposed spermatozoa and disintegrate, unless proteasomal inhibitors are included in coincubation mixture. The flow cytometric histograms of 5,000 cells/events per sample show an average from two replicates for the percentage of highly fluorescent cells (%-value; mean±SE) corresponding to the shadowed area of the histogram, and the median fluorescence of all flow cytometric events in the entire sample (M-value; mean±SE). Histograms of relative PNA-fluorescence are shown separately for gated acrosomal shrouds detached from sperm cells (left histogram column) and for the entire sample, including sperm cells and detached acrosomal shrouds (right histogram column). (**a**) The relative fluorescence of PNA is low prior to capacitation, in flow cytometer and by epifluorescence, because the OAM is concealed under sperm plasma membrane and not exposed for PNA-binding. (**b**) Fluorescence increases during capitation because OAM becomes exposed in the capacitated spermatozoa [2]; yet other spermatozoa undergo spontaneous acrosomal exocytosis. (**c**) Fluorescence level retreats back after sperm-ZPP coincubation under control conditions (only vehicles for proteasomal inhibitors were added) because the acrosomal shrouds detach from the spermatozoa and disintegrate. Most spermatozoa in this treatment show only the residual PNA binding to the newly exposed inner acrosomal membrane of the exocytosed spermatozoa (arrowheads); other spermatozoa are still in the process of exocytosis (arrow). (**d**) In the inhibitor group, fluorescence is even higher than in capacitated spermatozoa because proteasomal inhibition prevents the disintegration of acrosomal shrouds, whether still attached to sperm heads (arrow) or detached (arrowheads).

Binding of soluble ZPC protein to capacitated spermatozoa in our co-incubation system was readily detectable by immunofluorescence with antibodies specific to porcine sperm receptor glycoprotein ZPC (**Fig. 2a**). This binding was not affected by proteasomal inhibitors, yet the proteasomal inhibitors altered the ZP-protein-induced AE and resulted in the retention of the acrosomal shrouds on many spermatozoa (**Fig. 2a**). The observation that the inclusion of proteasomal inhibitors prevented the detachment and/or the disintegration of acrosomal shrouds from the ZPP-exposed sperm heads was further supported by the experiments showing lesser presence of acrosome-derived proteasomes in the supernatants in sperm-ZPP fractions coincubated in the presence of proteasomal inhibitors (**Fig. 2b**). Sperm acrosomal status and formation of the acrosomal shrouds upon co-incubation were monitored by flow cytometry of live, ZPP-exposed and control spermatozoa in which the acrosomes were labeled with fluorescently-conjugated, outer acrosomal membrane-binding lectin PNA [23] (**Fig. 2c-e**). Patterns of acrosomal shroud labeling with PNA and their flow cytometry-measured fluorescence intensities are shown in **Fig. 3 a-d**.

### Isolated Sperm Proteasomes Degrade Sperm Receptor ZPC in a Cell-Free System

**Figure 4:**
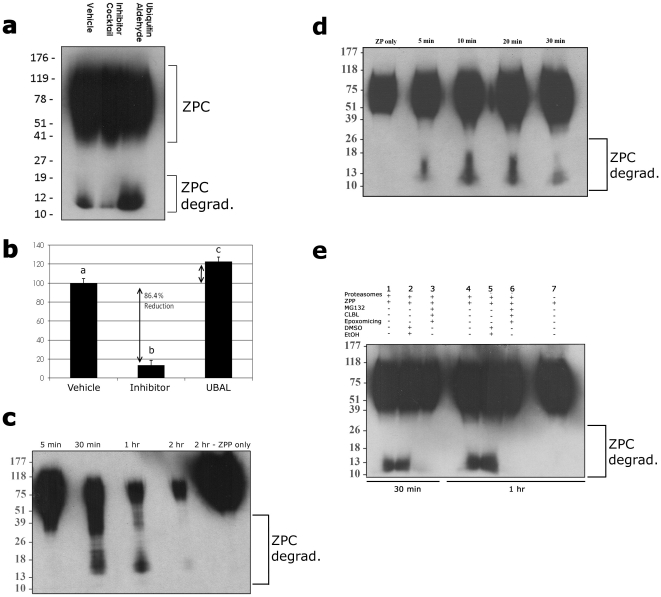
Proteolysis of ZPC in the presence of isolated sperm acrosomal proteasomes is similar to that in live sperm-ZPC coincubation-fractions. (**a**) Western blot of coincubated fractions with anti-ZPC antibody. After two-hours of ZPP-sperm proteasome co-incubation, a familiar degradation product appears (vehicle lane), which is reduced by the addition of proteasomal inhibitors (Inhibitor Cocktail lane), and amplified by the stimulation of proteasomal activity with ubiquitin aldehyde (UBAL lane). (**b**) Densitometry data from three replicates (lower panel) averaged 86% reduction (p<0.002) in the presence of proteasomal inhibitors, and a 23% acceleration (p<0.01) of proteasomal proteolysis with UBAL. (**c**) Time-lapse Western blotting of ZPC, revealing the progress of ZPC degradation during zona-protein coincubation with sperm proteasomes. No degradation products were observed in ZPP preparation incubated for 2 h without addition of isolated proteasome (last lane). (**d**) Thirty-minute time lapse of sperm-ZPC coincubation, revealing the formation of degradation product as early as 5 min. after ZPC-proteasome mixing. (**e**) Replicate of the two-hour time lapse experiment with isolated sperm proteasomes and solubilized zona proteins (ZPP), with/without addition of proteasomal inhibitor cocktail (MG132, CLBL & Epoxomicin), which eliminated the degradation of ZPC at 30 min (lanes 1–3) and 1 hr (lanes 4–7) after the onset of coincubation. Vehicles (DMSO, EtOH; lanes 2 &5) had no effect on degradation of ZPC; ZPP incubation for up to 1 hr without addition of proteasomes (lane 7) did not produce a detectable degradation product.

To ascertain that the observed ZPC proteolysis was specifically due to the activity of sperm proteasomes, purified proteasomes were isolated from boar spermatozoa (see proteasome characterization data, **Fig. S2**) and incubated with solubilized ZP-proteins. The reaction was supplied with energy in the form of ATP which is abundantly present and available to proteasomes in the intact boar spermatozoa [24], but has to be supplied externally for the sustenance of isolated proteasomes. The pattern of ZPC degradation by the sperm-derived proteasomes was very similar to that observed after ZPP co-incubation with whole spermatozoa (**Fig. 4** **a**). Also similar to whole sperm coincubation with ZP-proteins, proteasomal inhibitors reduced ZPC-degradation by isolated sperm proteasomes by 86%, compared to vehicle group (**Fig. 4** **b**). Furthermore, acceleration of proteasomal proteolysis with UBAL increased the density of the 12 kDa ZPC-degradation product by 23%, compared to proteasomes alone (**Fig. 4** **b**). A time-lapse Western blotting experiment revealed a progressive degradation pattern with a most prominent degradation product observed at 30 min. of coincubation (**Fig. 4** **c**). This degradation product was already visible after first 5 min. of coincubation (**Fig. 4** **d**), suggesting that ZPC degradation by sperm proteasomes occurs very rapidly. No degradation products were observed in ZPP preparations incubated for up to 2 h without the addition of isolated proteasome (**Fig. 4** **c, d**). Similar to two-hour incubation, the proteasomal inhibitor cocktail prevented the degradation of ZPC at 30 min and 1 hr after the onset of coincubation (**Fig. 4** **e**).

### Porcine Zona Proteins are Ubiquitinated

**Figure 5:**
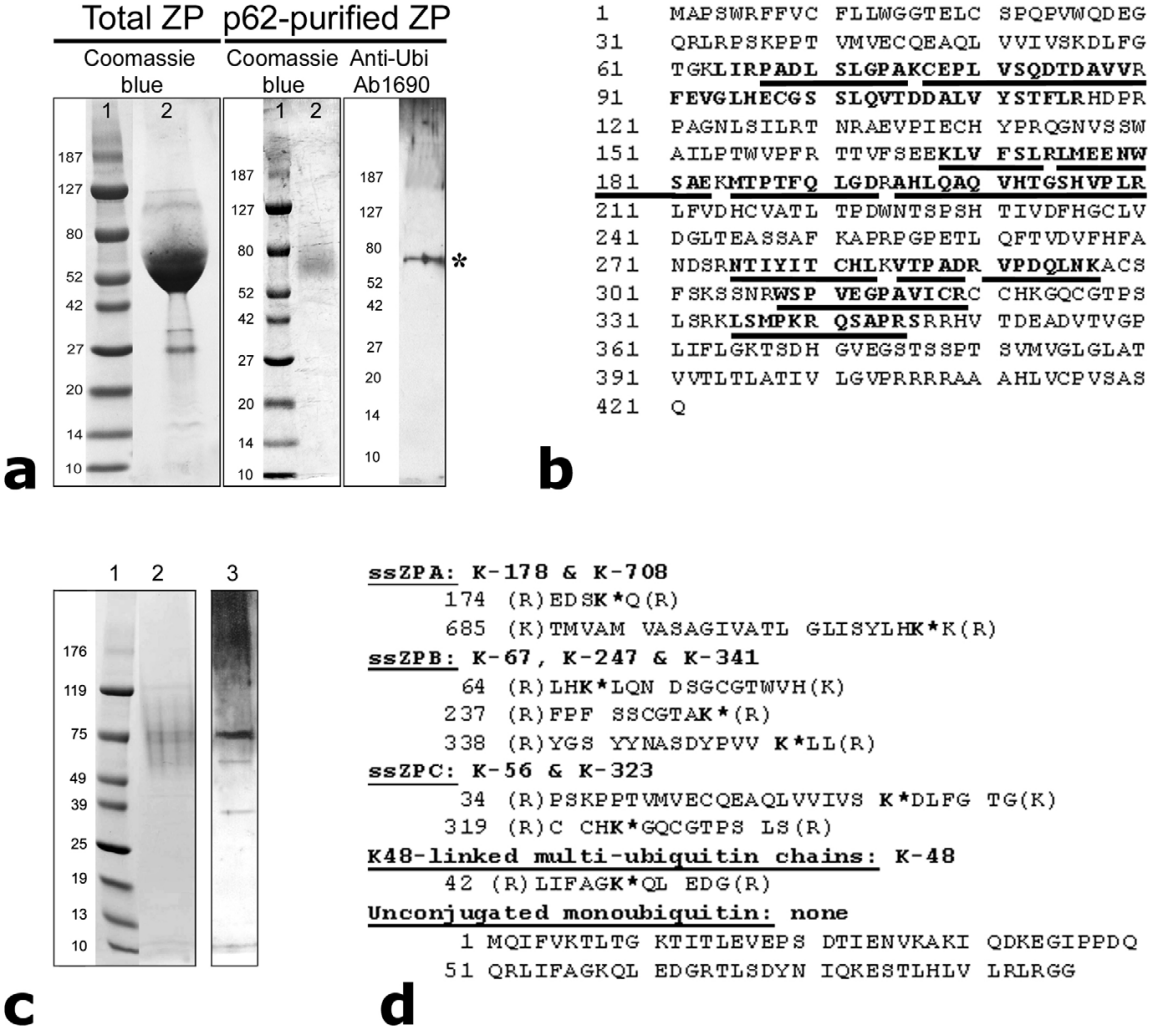
Porcine zona pellucida proteins are ubiquitinated prior to fertilization. (**a**) Zona pellucida fragments separated from minced porcine ovaries were solubilized (Left; Coomassie-stained PAGE gel-lane 2; lane 1 = protein markers), and subjected to affinity purification of ubiquitinated proteins using the recombinant UBA domain of ubiquitin-binding protein p62 (center; PAGE gel, lane 2). The protein band between 70–80 kDa was reactive to anti-ubiquitin antibodies (Right; Western blot). (**b**) This band was excised and identified by MALDI-TOF MS as porcine sperm receptor component ZPC (identified fragments are underlined). (**c**). Ubiquitinated proteins from solubilized zonae from preselected, morphologically normal porcine metaphase-II oocytes were affinity purified on p62 matrix (lane 2; PAGE) and showed immunoreactivity to anti-ubiquitin antibodies (lane 3; Western). (**d**) Soluble proteins isolated by p62 affinity-purification were subjected to Nanospray LC-MS/MS spectroscopy adjusted for Gly-Gly modification, a fingerprint of ubiquitinated internal Lys-residues. Gly-Gly modifications were observed on all three components of porcine ZP, including ZPA, ZPB and ZPC, and in the positive control, the K-48 linked multi-ubiquitin chains, but not in the unconjugated monoubiquitin.

The hypothesis that sperm proteasomes are responsible for digesting a fertilization slit assumes that the target ZP-protein is already tagged with ubiquitin prior to fertilization, i.e. during oogenesis, as observed in the porcine ovarian follicles [16] and also in the unfertilized sea urchin eggs [17]. This pattern of zona pellucida/vitelline membrane ubiquitination would be an alternative to ubiquitination of the sperm receptor by ascidian sperm exudates, observed during ascidian fertilization [18]. Therefore, we subjected the isolated porcine ZP protein to modified proteomic analysis capable of distinguishing the Gly-Gly-modification [25] characteristic of ubiquitinated proteins. Initially, the solubilized ZP proteins isolated from porcine ovaries [26] were subjected to affinity purification of ubiquitinated proteins using the recombinant UBA domain of ubiquitin-binding protein p62 (**Fig. 5 a**). A distinct protein band reactive to anti-ubiquitin antibodies was detected by Western blotting and the corresponding band from a PAGE gel was identified by MALDI-TOF mass spectroscopy as porcine sperm receptor component ZPC (**Fig. 5 b**), known to form the porcine sperm receptor complex by hetero-oligomerization with ZPB [3]. Compared to the ZPP band density in the initial protein load prior to affinity purification, the purified ubiquitinated protein band was relatively weak. This was likely due to low yield of the highly specific, low affinity purification procedure, or to the possibility that only some of the ZPP protein molecules are modified by ubiquitination. To identify the ubiquitinated internal lysine sites on porcine ZP proteins, we solubilized zonae from preselected, morphologically normal porcine metaphase-II oocytes matured *in vitro* (**Fig. 5c**) and subjected them to Nanospray LC-MS/MS spectroscopy. Database searches were adjusted to identify peptides containing lysine residues modified with a covalently attached di-aminoacid (Gly-Gly), which is a fingerprint of ubiquitinated internal Lys-residues [25]. We found Gly-Gly modifications on all three components of porcine ZP, including ZPA, ZPB and ZPC (**Fig. 5d**). Such modifications were also identified in the positive control, the K-48 linked multi-ubiquitin chains, but not in the unconjugated monoubiquitin, in which ubiquitinated Lys-residues are not expected (**Fig. 5d**). Altogether, two different proteomic techniques found that ZPC was ubiquitinated in ZP proteins prepared using different methods.

### ZPC Colocalizes with Ubiquitin on the Zona and in Corona Radiata Cells

**Figure 6:**
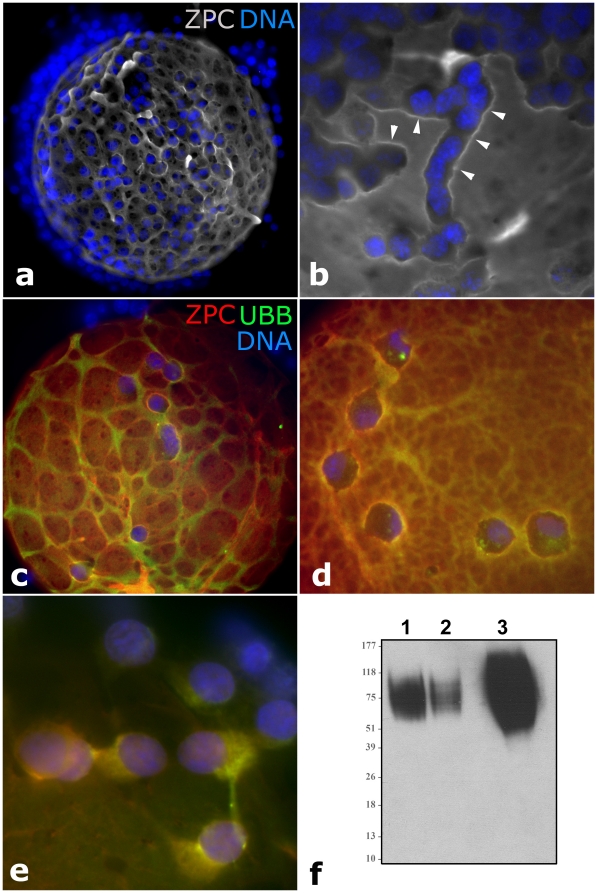
Patterns of zona pellucida deposition and ZPC-ubiquitin colocalization in porcine ocyte-cumulus complexes isolated from small antral follicles. (**a, b**) Accumulation of ZPC (gray) in ridges (arrowheads) adjacent to zona-adhering corona radiata cells (blue  =  nuclei stained with DAPI). (**c-e**) Colocalization of ZPC (red) and ubiquitin (green) in the zona pellucida (c, d) and in the cytoplasm of corona radiata cells (e); DNA was counterstained with DAPI (blue). Western blotting of ZPC protein in the isolated cumulus/corona cells from 60 oocyte cumulus complexes (lane 1), in 60 zona-free oocytes (lane 2) and in soluble zona proteins isolated from 60 oocytes (lane 3).

Rather than being a homogeneous layer of extracellular matrix, porcine zona labeled with anti-ZPC antibody exhibits a bone-marrow like structure. A crisscrossed pattern of ECM cords on the surface appears to be due to uneven deposition of zona proteins in the gaps between corona radiata cells covering the zona inside an ovarian follicle (**Fig. 6 a, b**). It was in these cords that a most intensive overlap was observed between immunolabeled ubiquitin and ZPC protein (**Fig. 6 c, d** **; and Fig. S3**). Overlapping accumulation of ZPC and ubiquitin was also observed in the cytoplasm of corona radiata cells surrounding the oocytes isolated from small/growing antral follicles (**Fig. 6 e**). Western blotting confirmed the presence of ZPC protein in both the zona free oocytes and in the isolated corona radiata cells (**Fig. 6 f**).

### Proteasomal Inhibitors Protect the Proteins of Acrosomal Shroud from Degradation

**Figure 7:**
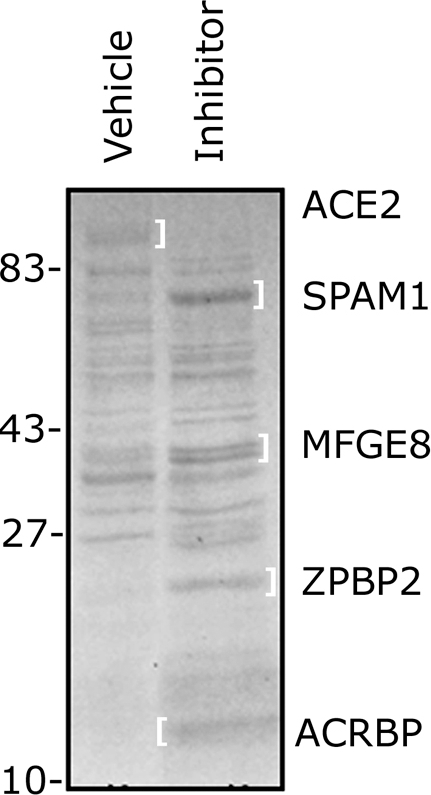
Proteasomal inhibitors protect sperm acrosomal surface-associated proteins from degradation during ZPP-induced acrosomal exocytosis. Sperm-ZPP supernatants containing acrosomal shrouds and soluble/shroud bound zona proteins after 2 h of sperm-ZPP coincubation were separated on 1D PAGE gels. Bands that differed between vehicle control-fraction (left lane) and inhibitor fraction (right lane) were excised and subjected to LC-MS/MS identification. All of the identified proteins are known to be acrosomal components. ACE2 was the only protein diminished in the inhibitor-exposed fraction.

In addition to actual degradation of the zona proteins, acrosomal proteasomes play a role in the process of acrosomal exocytosis [27], [28], [29]. To determine if the proteasomal inhibitors were altering the proteolytic processing of acrosomal proteins in the capacitated spermatozoa stimulated by soluble ZP-proteins, we used 1D PAGE gels to separate the proteins from supernatants containing acrosomal shrouds and soluble zona proteins. Based on differences in band patterns of fractions incubated with proteasomal inhibitors or with control vehicle solutions, several bands unique to these respective treatments (**Fig. 7**), were excised and subjected to LC-MS/MS identification. Among the identified proteins that were protected from proteasomal degradation by specific proteasomal inhibitors were Sperm Adhesion Molecule 1 (SPAM1), MFGE8 (aliases lactadherin, SED1, SP47), Zona Pellucida Binding Protein 2/ZPBP2 (alias IAM38) and a fragment of Acrosin-Binding Protein ACRBP (alias SP32). The Angiotensin I Converting Enzyme 1 Isoform 2/ACE2 was more abundant in vehicle group (**Fig. 7**)**.** Identified tryptic fragment sequences are shown in **Fig. S4**). The same results were obtained in a repeated experiment. Most likely, these structural proteins of the acrosomal cap are degraded during AE to allow for the acrosomal membrane fusion and vesiculation of the outer acrosomal membrane.

### Isolated Sperm Proteasomes Degrade Intact Zonae *In Situ*

**Figure 8:**
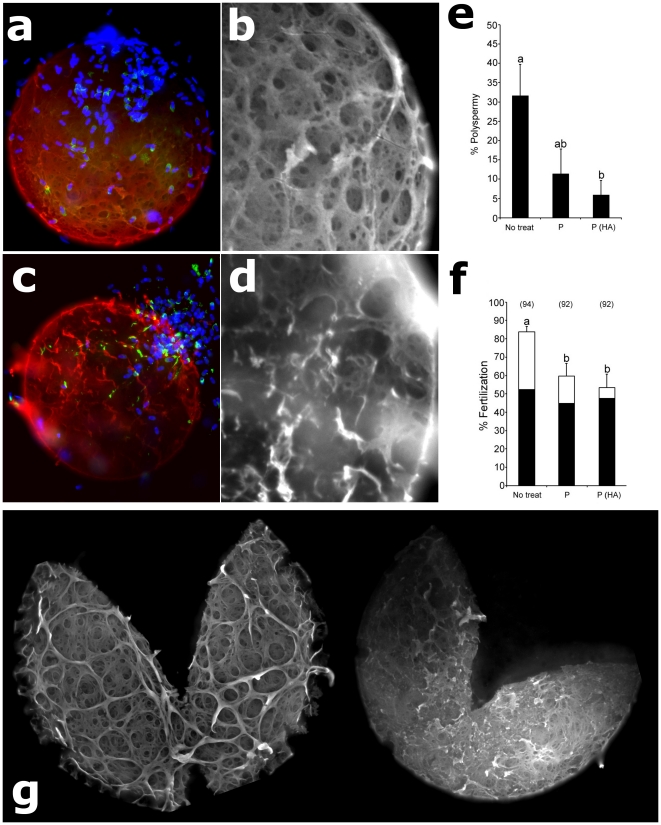
Degradation of Intact Oocyte Zonae by Isolated Sperm Proteasomes. Maturing ova were incubated with purified sperm proteasomes for four hours with non-activated (**a, b**) or heat activated (**c, d**) sperm proteosomes, fertilized in vitro, and processed with anti-ZPC antibody (red), DNA stain DAPI (blue) and acrosomal shroud marker - lectin PNA-FITC (green). Note sperm detachment, and a striking abrasion and loosening of the zona in the active proteasome-treated ova (**c,** **d**). Preincubation with active proteasomes coincides with a reduced rate of polyspermic fertilization after IVF (**e, f**). IVF experiment was repeated three times, with total oocyte numbers shown above each column in panel **f**. Control (**g**; left) and proteasome treated (**g**; right) ova were also processed with anti-ZPC antibody immediately at the end of the 4 h coincubation, causing a pattern of zona digestion and abrasion (right zona) similar to that seen in IVF ova pre-treated with active proteasomes.

The above data on proteasomal degradation of solubilized ZPC trigger expectation that isolated proteasomes would also degrade intact zonae surrounding mature, fertilization competent ova. Thus, we developed an assay in which in vitro maturing pig ova are incubated with purified, heat-activated sperm proteasomes during the last four hours of *in vitro* maturation (total 44 h of IVM). Heat activation for 20 min at 55°C is an efficient method to activate purified proteasomes [30], [31]. After 4 h of coincubation, oocytes were washed, fertilized by standard IVF and processed with antibody against ZPC (as also used for Western blotting/densitometry; see **Fig. S1**), DNA stain DAPI and acrosomal shroud marker - lectin PNA-FITC. This coincubation of *in vitro* maturing ova with purified, heat-activated sperm proteasomes caused zona digestion, resulting in a striking abrasion and loosening of the zona (**Fig. 8** **a-d**). This treatment coincided with a reduced rate of polyspermic fertilization (**Fig. 8** **e**), which commonly occurs in porcine IVF systems. Strikingly, the rate of monospermic fertilization remained constant at ∼50%, while the polyspermy rate was reduced from >30% to only 7% (**Fig. 8** **f**). Control ova were incubated for 4 h with proteasomes that had only basal activity (they were not heat activated). Besides examining the fertilization rates of the proteasome-treated ova, groups of control and treated ova were also processed with anti-ZPC antibody immediately at the end of the 4 h coincubation. Patterns of zona digestion and abrasion similar to those seen after IVF were observed in unfertilized ova exposed to heat-activated proteasomes (**Fig. 8** **g**). Thus, it appears that the observed zona digestion was not occurring only after IVF. Altogether, this experiment demonstrates the ability of isolated sperm proteasomes to digest an intact pig zona, in addition to digesting soluble zona proteins.

## Discussion

We show here for the first time that mammalian sperm proteasomes degrade ZP proteins in a coincubation system relevant to fertilization. Proteasomes purified from the acrosomal fractions as well as those carried by capacitated motile spermatozoa were efficiently inhibited by a trio of specific proteasomal inhibitors including MG 132, CLβL, and Epoxomicin. Such an inhibition protected the solubilized sperm receptor glycoprotein ZPC from degradation during its coincubation with sperm cells or purified sperm proteasomes. Furthermore, ZPC degradation by sperm proteasome was accelerated by ubiquitin-aldehyde, a modified ubiquitin protein that has a stimulatory effect on proteasomal proteolysis by blocking the activity of ubiquitin C-terminal hydrolases [32]. The respective effects of proteasomal inhibitors and ubiquitin-aldehyde on ZPC degradation in a cell free system bear striking similarity to their action during *in vitro* fertilization. Proteasomal inhibitors block *in vitro* fertilization completely at the stage of sperm-ZP penetration [16]. Ubiquitin-aldehyde, in turn, stimulates fertilization overall, and causes a massive polyspermic fertilization in vitro [33]. These results provide strong support for the enzymatic hypothesis of sperm penetration through the mammalian ZP. While the entire act of fertilization may not be accounted for by the sperm associated proteolytic activity, one cannot dismiss the fact that enzymes found in the sperm acrosomal matrix are required for sperm penetration through the ZP [9]. Not only are ZP proteins degraded by the sperm proteasomes in a coincubation system, but several acrosomal proteins are subject to proteasomal degradation as well. This finding explains why proteasomal inhibitors have the ability to stop acrosomal exocytosis at an early stage of its signaling cascade, i.e. upstream of calcium influx in the acrosome [27], [34].

The requirement of proteasomal proteolysis during sperm-ZP interactions is likely two-fold. First, the sperm acrosomal proteasomes degrade the sperm receptor protein on the ZP. This likely loosens up the structure of the ZP and is conducive to mechanical, motility-driven sperm-ZP penetration as well as to action of proteases other than 26S proteasome. Present data on proteasomal degradation/loosening of the intact porcine zonae support this interpretation. Second, sperm acrosomal proteasomes seem to degrade proteins associated with acrosomal membranes. This may lead to the formation and then to eventual breakdown of the sperm acrosomal shroud, which we have traced by flow cytometry. The formation of the acrosomal shroud may initially create a complex microenvironment consisting of zona-bound membrane vesicles, viscous acrosomal matrix and soluble as well as membrane anchored proteases and glycosidases. These enzymes may be held in place by the shroud and necessary for the digestion of the ZP surface around the leading edge of the zona-bound sperm head. Alternatively, proteasomal degradation of acrosomal zona-binding proteins, such as ACE2 and ZPBP2, observed during sperm-ZPP coincubation, could serve to terminate primary sperm-ZP binding as the ZP-bound sperm head starts to move forward and penetrate deeper into ZP. Both ACE2 and ZPBP2 have been implicated in the initial binding of the sperm acrosome to ZP [35], [36].

The in vitro ZPC degradation assay was performed in the presence of extrinsic ATP. In somatic cells, ATP is almost exclusively intracellular. However, spermatozoa are sensitive to external ATP. Adding 2.5 mM extracellular ATP to the capacitated bull spermatozoa stimulated acrosome reaction and increased the percentage of acrosome-reacted spermatozoa [37]. Authors suggested that extracellular ATP activates a signaling cascade of acrosome reaction via ATP purinoreceptor of P_2y_ type. The addition of extracellular ATP also improved IVF results in humans [38] and increased linear sperm motility in mice [39]. The presence of extracellular, sperm-surface associated ATP was indirectly demonstrated in a free-spawning animal, sea urchin *Pseudocentrotus depressus,* via depletion of cell surface ATP by *Solanum tuberosum* apyrase, added directly to sea water with gametes. Apyrase is a large, 49 kDa enzyme that cannot penetrate the cellular plasma membrane. Consequently, it is expected to interact exclusively with the extracellular and cell-surface associated ATP when added in the fertilization medium. Therefore, similar to sea urchin fertilization, purified apyrase blocked fertilization in the porcine IVF system [24]. In these studies, sperm-surface associated ATP was measured directly by a fluorometric assay. A pool of extracellular/sperm surface ATP was found, associated with the acrosomal surface. It is plausible that ATP associated with sperm acrosomal surface can be utilized by the sperm proteasomes during fertilization. Although ATP is not required for substrate degradation in the 20S proteasomal core, it is required for maintaining the integrity of the 19S proteasomal regulatory complex, which contains six subunits with ATPase activity. In accordance, ATP depletion from boar sperm surface altered the integrity of sperm proteasomes, manifested by an altered proteasomal subunit migration on SDS-PAGE [24].

Extracellular function of ubiquitin-proteasome pathway in mammalian gametes is unexpected in the context of general cell biology, yet the concept of extracellular ubiquitin systems has been gaining acceptance (see Reviews by [14], [40]). Zona protein synthesis and secretion differs from species to species. While oocytes seem to be uniquely responsible for zona protein secretion in the mouse [41], localization of ZPA, ZPB, and ZPC mRNA changes with follicular stage in other mammals. Production of the canine ZP glycoprotein mRNAs starts from the oocyte cytoplasm in the primordial follicle, but gradually shifts toward adjacent cumulus cells in the growing follicle [42]. Rabbit ZP glycoprotein ZP1 mRNA has been localized to both the oocyte and granulosa cells in primary follicles [43]. In the case of porcine oocyte-cumulus complex, both the oocyte and zona-adjacent cumulus/corona radiata cells express ZP-encoding genes [44]. Since zona deposition likely requires a high rate of ZP-protein secretion, it is possible that the zona proteins become ubiquitinated in the secretory pathway. The Endoplasmic Reticulum Associated Protein Degradation (ERAD) system is responsible for ubiquitination and proteasomal degradation of malfolded proteins in the ER, assuming that ubiquitination occurs during substrate translocation through ER membrane and proteasomal degradation occurs in the cell cytosol (reviewed by [45], [46]). Given the gaps in knowledge of ERAD mechanisms and possible limitations of ERAD efficiency in clearing defective proteins, it is possible that ubiquitinated proteins could be secreted. As such, ubiquitinated ZP-proteins could escape degradation in the ER and be deposited in the oocyte zona. The observation that genes encoding ZP proteins appear to be expressed predominantly in the cumulus cells during the late stages of porcine oocyte/follicle growth [44] agrees with the overlapping accumulation of ZPC and ubiquitin on the pig zona surface, and specifically in the cumulus cell cytoplasm and in the gaps between zona-adhering cumulus/corona radiata cells (see Fig. 6). Alternatively, zona proteins could be ubiquitinated after being secreted, as ubiquitin is present at a high concentration in the ovarian follicular fluid [47]. Finally, ECM components other than zona glycoproteins are secreted by porcine oocyte and cumulus cells and bind to zona surface [48]; such ECM components could also be ubiquitinated and subject to degradation by the sperm proteasomes during fertilization.

There is now evidence that both the sperm acrosome (reviewed in [49]) and spermatid acrosomal cap [50], [51] contain proteasomes. While the association of proteasomes with an organelle that ultimately undergoes exocytosis may appear unusual in the context of somatic cells, it is not uncommon for gametes to utilize cellular pathways such as the secretory pathway in an unorthodox manner. The sperm acrosome itself is a fitting example. Acrosomal membranes (outer and inner) and matrix are derivatives of trans-Golgi in a spermatid, but the secretory vesicles detaching from the spermatid are not docked to the inner face of the plasma membrane [52], as would be the case in somatic cells. Instead, they are directed toward the nucleus where they attach to a specialized structure referred to as subacrosomal perinuclear theca, or acroplaxome. At this stage, the anchoring of proteasomes is already observed on the cytoplasmic face of the membranes of the future acrosomal cap [50].

While both *in vitro* fertilization [16] and ZP-protein degradation (this study) is efficiently blocked by proteasomal inhibitors, consideration should be given to other proteases that may facilitate acrosomal exocytosis and sperm-zona penetration in mammals. Conventional protease inhibitors have been shown to prevent sperm penetration through the zona pellucida [6], though some of this inhibition may be due to an undetected inhibitory effect on sperm proteasomes. However, several acrosomal enzymes not related to proteasome, such as serine proteases ACR (acrosin) and PRSS21 (TESP5) may participate in the processing of the sperm acrosomal matrix and sperm penetration through the egg zona, resulting in fertilization [16], [53], [54]. For instance, the male *Acr-*null mice are fertile but exhibit delayed AE during fertilization *in vitro* [12] and the *Acr-Prss21* double-knockout mice are subfertile *in vivo,* and their sperm are unable to fertilize eggs *in vitro* [13].

Collectively, our results validate the hypothesis that the sperm acrosome-borne 26S proteasomes recognize and degrade ubiquitinated zona pellucida proteins during mammalian fertilization. Such data point to evolutionary conservation of the proteasome-dependent mechanism adopted by animal spermatozoa for vitelline coat penetration: The sperm receptor on ascidian egg coat, called HrVC70, that is degraded by sperm proteasomes during ascidian fertilization is in fact the ascidian homologue of mammalian sperm receptor ZPC [18], shown to be targeted for proteasomal degradation in our coincubation system. Our findings have many implications for assisted fertilization in humans and animals. Simple, rapid methods for measuring of proteasomal activities in human and animal sperm samples using specific fluorometric substrates (e.g. [24], [33]) could be adapted for male fertility testing in farm animals and for diagnostics of human male infertility. Since the proteasomes are uniquely exposed on the cell surface in human, pig, mouse and ascidian spermatozoa [14], [18], [34], [55], [56], they could be targeted to elicit a contraceptive effect by antibodies and proteasome-inhibiting molecules other than cell-permeant proteasomal inhibitors, which would likely exhibit side effects. Coincidentally, anti-proteasome antibodies are found among anti-sperm antibodies present in seminal plasma of men suffering from autoimmune infertility [57]. Searching for possible immunocontraceptive targets in human spermatozoa by using yeast two-hybrid system, Naz and Dhandapani [19] recently identified the ubiquitin-binding, proteasome-associating protein UBAP2L as the most likely binding partner of human ZPC protein. Furthermore, antibodies against UBAP2L inhibited human sperm-zona binding in a hemizona assay [19].

## Materials and Methods

### Zona Pellucida Collection

Oocytes were aspirated using an 18 gauge needle and a 10 ml syringe from ovaries collected from a slaughterhouse (Farmland Foods, Milon, MO). Oocytes with cumulus cells were placed in 500 µl of TCM-199 media containing; FSH, LH, EGF, and follicular fluid. The oocytes remained in this media for 22 hrs, at which time they were moved into media containing no hormones for an additional 22 hrs. After maturation the cumulus cells were removed by agitation in TL-Hepes containing 0.1% polyvinylacohol (PVA) and 0.5% hyaluronidase. Oocytes were exposed to 10 µl TBS (213 mM NaCl, 50 mM Tris) pH 2.0. This solubilized the ZP and 10 µl of TBS pH 8.0 was then added to the oocytes to neutralize the solution. The solubilized zona pellucida proteins (ZPP) were collected from the oocytes, placed in 500 µl tubes and stored in a −20°C freezer until required.

### Sperm Capacitation and Fertilization *In Vitro*

All studies involving vertebrate animals were completed under the strict guidance of ACUC protocol number #A3394-01, approved by the Animal Care and Use Committee of the University of Missouri. Fresh boar spermatozoa were collected on the day of co-incubations. The collected spermatozoa were measured into a 15 ml Falcon tube and spun at 350×g in a Fisher Scientific Centrifuge for 5 min to remove the seminal plasma. The supernatant was removed and the pellet was resuspended in 14 ml TL-Hepes-PVA and spun at 350×g for 5 min. The sperm pellet was then resuspended in 6 ml TL-Hepes-PVA. In a new Falcon tube 30 million washed spermatozoa were added to 12 ml of capacitation media containing TL-Hepes, 11 mM glucose, 5 mM pyruvic acid, and 20 mg/ml BSA and capacitated for 6 hrs at 38.5°C before being exposed to solubilized ZP. For IVF experiments, oocytes were maturated as described above, and fertilized with 500,000 sperm/ml according to a standard protocol [24].

### Sperm-ZPP Coincubation Experiments

Upon the completion of capacitation, the spermatozoa were spun at 300×g for 5 min, the supernatant was removed and the pellet resuspended in 13 ml TL-Hepes-PVA. The solution was spun again at 300×g for 5 min and the pellet was resuspended in 6 ml TL-Hepes-PVA prior to being analyzed for final sperm concentration. New 15 ml Falcon tubes were marked for the individual groups, to these tubes 10,000 spermatozoa were added and then spun at 300×g for 5 min. After removing the supernatant, appropriate amounts of TL-Hepes-PVA was added to the sperm pellets to bring the total reaction volume to 30* µ*l. Following the addition of TL-Hepes-PVA, the groups received either the Inhibitor cocktail containing 100* µ*M MG 132, 100* µ*M CLβL and 100* µ*M Epoxomicin, or vehicle solution containing 100 *u*M EtOH, and 100 *u*M DMSO, or ubiquitin-aldehyde at 5* µ*g/ml (all inhibitors were purchased from Enzo-Biomol; Plymouth Meeting, PA). Once the treatments were added to the sperm pellets, solubilized ZP protein purified from 100 oocytes was added and mixed with the sperm pellets. The incubations continued for 2 hrs at 38.5°C and 5% CO_2_ at which time the solutions were removed from the Falcon tubes, placed in 1.5 ml Eppendorf tubes and centrifuged at 300×g for 5 min in the Sorvall Biofuge Fresco centrifuge. After centrifugation the supernatants and pellets were separated and kept for analysis of proteins by Western blotting.

### Flow Cytometric Analysis of Sperm Acrosomal Status

The effect of soluble zona proteins and proteasomal inhibitors on sperm acrosomal status and formation of the acrosomal shrouds was assessed by flow cytometry with fluorescently labeled lectin PNA. This lectin binds with differing affinities to outer acrosomal membrane in capacitated and acrosome-reacted spermatozoa, as well as to acrosomal membrane vesicles of the detached acrosomal shrouds. Analysis was performed on a Guava EasyCyte Plus flow cytometer (Guava Tech, Hayward, CA) operating on a Cytosoft platform (IMV Technologies, L'Aigle, France). Briefly, 2.0 µl of sperm in solution per treatment were mixed with 0.8 µl of PNA-Alexa488 (Molecular Probes-Invitrogen) and 197.2 µl of TL-Hepes medium in a 1.5 ml Eppendorf tube, and incubated for 30 min at room temperature. Upon completion of incubation, the reaction mixtures were loaded onto a 96 well plate and analyzed by Guava using the following instrument settings: Forward Scatter Gain - x8, Side Scatter – 500 V, Green Fluorescence – 413 V, Yellow – 403 V, Red – 701 V, and medium sample flow rate – 0.59 µl/s. All samples were acquired in duplicate and the experiment was replicated twice.

### Sperm Proteasome-Purification and Coincubation with ZPP

To isolate proteasomes, 30 ml of freshly collected boar semen were washed 3 times in PBS by centrifugation in a Fisher Scientific centrifuge at 350×g for 5 min. The washed semen samples were sonicated with a Branson Sonicator (Branson Ultrasonic Corp. Danbury, CT) at 30% intensity for 1 min in cold TBS (104 mM NaCl, 96 mM Tris, pH 7.4) supplemented with 1 mM DTT, 1 mM EDTA, and 10% glycerol. After sonication the contents were separated into two 15 ml Falcon tubes and centrifuged at 300×g for 10 min and the supernatants were collected. The supernatants were then centrifuged in the Beckman centrifuge with 50.2 Ti-rotor at 100K×g for 2 hrs. The clear supernatants were collected and concentrated by additional centrifugation in 10K Centricon tubes (Millipore, Billerica, MA) at 4,000×g until the total volume of the supernatant fractions were reduced down to 1.5 ml. The extracts were then aliquoted and stored at −80°C. Upon being thawed, 5* µ*l of purified proteasomes were incubated with solubilized ZP protein from 100 oocytes either in the presence of the vehicles or the inhibitor cocktail as previously stated. The treatment groups were supplemented with 5* µ*M ATP.

### Antibodies

The monoclonal antibody MA-467, reactive with the N-terminal domain of the peptide backbone of porcine ZPC glycoprotein and devoid of reactivity with pig ZPB by Western blotting, was generated and validated as described previously [20], [21], [22]. The ascites produced in inbred BALB/cj mice was used at 1∶2,000 dilution. It showed no reactivity with the related ZPB protein in ELISA and Western blotting. It also reacted with deglycosylated, reduced, and carboxyamidomethylated (RCM) forms of porcine ZPC, both in ELISA and Western blotting, thus suggesting that it reacts with the polypeptide backbone of ZPC. The epitope mapping studies using tryptic fragments of porcine ZPC followed by sequencing and mimotopes strategy revealed that MA-467 recognized the peptide corresponding to QPVWQDEGQRLR sequence (aa 23-34) of ZPC that is in the N-terminal part of the mature, secreted porcine ZPC. Using single amino acid substitutions, the minimum binding motif was mapped to WQDE of ZPC. MA-467 also inhibited boar sperm binding to zona enclosed porcine oocytes.

Further characterization of the MA-467 revealed that prior incubation of porcine eggs with this antibody significantly delayed the time required for zona lysis by Trypsin. In Western blotting, this antibody reacted with 18 kDa band of recombinant ZPC digested with Lys-C, and with the 37 and 30 kDa bands of recombinant ZPC digested with elastase. It is thus reasonable to assume that this antibody will recognize the N-terminal fragments of porcine ZPC protein produced by proteasomal proteolysis.

Mouse IgG against 20S proteasomal core subunits α1-7 (PW 8195; Enzo-Biomol) recognizes a common motif found in subunits α1 (PSMA6), α2 (PSMA2), α3 (PSMA4), α5 (PSMA5), α6 (PSMA1) and α7 (PSMA3). An anti-ubiquitin rabbit polyclonal antibody AB1690 (Chemicon, Rosemont, IL) was raised against KLH-conjugated full-length ubiquitin protein. Mouse anti-ubiquitin antibody KM691 was purchased from Kamiya Biomedical Company, Seattle, WA.

### Western Blotting and Densitometry Analysis

The acrosomal shrouds containing ZP-bound proteins were separated from the sperm pellet by centrifugation at 500×g for 5 min. The supernatant was boiled at 95°C for 5 min in 2X loading buffer containing; 100 mM Tris, 300 mM NaCl, 4% SDS, 10% β-mercaptoethanol, 40% glycerol, and Bromphenol Blue. Total loading volumes for the gel from the supernatants did not exceed 30 µl. Pellets from the coincubation experiments were boiled as previously stated and then centrifuged at 3,000×g for 10 min before loading 30 µl from each sample into a 4–20% gradient gel purchased from Lonza (Basel, Switzerland). Proteins were transferred on to PVDF membrane purchased from Millipore (Billerica, MA). Antibodies raised against ZP glycoprotein ZPC were used as primary antibodies at a 1∶2,000 dilution. Primary antibody incubation occurred overnight at 4°C, washing between antibodies was done using 1% nonfat dry milk in TBS with 0.1% Tween for 25 min. Secondary antibodies were anti-mouse IGg HRP antibody (Zymed) and were used at a 1∶20,000 dilution for 40 min at room temperature in 1% nonfat dry milk in TBS/Tween. After secondary incubation, a final washing was done using TBS/Tween solution for 35 min. Antibodies raised against β tubulin (TUBB) were used as a loading control to assure equal loading between samples, and used to normalize for densitometry. The membrane was incubated in a volume of 3 ml of chemiluminescent HRP substrate for 5 min prior to being exposed to Kodak film. Differences in protein bands were determined by densitometry analysis using the Kodak 1D Image Analysis Software and Kodak films developed on the Hope micro-max developer. Statistics were performed on all three replicates by an ANOVA table.

### 1D Gel Proteomics of Sperm Acrosomal Fractions Coincubated with ZPP

Protein bands were excised from 1D SDS-PAGE gels stained with Coomassie Blue, followed by in-gel digestion with trypsin (Promega Gold MS grade). Peptides were partially purified by C-18 (ZIP tip) peptide clean-up prior to analysis by MALDI TOF-TOF MS & MS/MS. These results were then referenced for similarity of known porcine proteins by using the Applied Biosystems' GPS Explorer software version 3.6 prior to being submitted for cross reference with the NCBInr mammalian protein database.

### Nanospray LC-MS/MS of Zona Proteins

LC/MS/MS was also performed by first trapping the in-gel digested peptides on a C-18 CapTrap (Michrom) in 2% acetonitrile for 10 min. Peptides were then eluted onto a 0.6×100 mm C-18 column (Agilent) using a linear 5–45% gradient of mass spectrometry grade acetonitrile (Sigma) in water (Burdick and Jackson, Honeywell) containing 0.1% formic acid (Fluka) over 80 min followed by 98% acetonitrile for 10 min and re-equilibration in 2% acetonitrile. Eluted peptides were analyzed using an LTQ ion-trap mass spectrometer running Excalibur 2.2 software. Excalibur was set to first collect a survey scan from 300 – 2000 Da followed by data-dependent scans on the top three ions. Dynamic exclusion was enabled and set for a repeat count of two, duration of 30 sec and a mass width of 1.0 Da. DTA generation parameters were set for a MW range of 500–4000, threshold of 200, precursor mass of 2.0 Da, and a minimum ion count of 20. MS/MS spectra were then searched against an NCBInr protein database using both Proteome Discoverer 1.0 (ThermoFinnigan) and Peaks 5.2 (Bioinformatics Solutions Inc.) *de novo* search programs. Peptide mass tolerance was set at 3.0 Da and product ion tolerance was set to 0.8 Da. Variable modifications were set to include oxidation of methionine and Gly-Gly addition to lysines, fixed modifications were set to include carbamidomethylation of cysteine residues. Minimum acceptable quality peptides were set by false discovery rates of 0.05 generated from reverse database searches. At this false discovery rate minimum acceptance criteria were: Xcorr = 1.5 sor 1+ charge, 2.2 for 2+ charge and 2.5 for 3+ charge.

### Immunofluorescence and Epifluorescence Microscopy

Samples of 10 µl were taken from each sperm treatment group following the 40 min incubation with lectin PNA. Samples were placed on Super Frost microscope (Fisher) slides and covered with 22×22 Premium cover slips (Fisher). Once the cover slips were in place, clear nail polish was used to seal the edges of the cover slips. Immunofluorescence double-labeling of ZPC (see antibody details above) and ubiquitin (antibody KM691) was performed in the oocytes and zygotes as described previously [58]. Slides were viewed on the Nikon Eclipse E800 microscope under the 40× magnification. Images were taken using the Nikon Cool Snap HQ camera and viewed through Metamorph Imaging Software Version 7.1.

## Supporting Information

Figure S1 **Immunolabeling of porcine ZP with anti-ZPC antibody MA-467, as also used for Western blotting.** A representative, mature, metaphase II-stage oocyte is shown on an optical cross section across the equatorial plane (**a**) and surface plane (**b**). An oocyte with a cracked ZP is also shown (**c**) to demonstrate the difference between the fluorescence intensity of the ZP and the ooplasm, exposed by the crack.
(TIF)

Figure S2 **Isolation and characterization of the sperm acrosomal proteasomes.** (**a**) Proteasomal activity of the purified sperm acrosomal proteasomes is demonstrated by time-dependent digestion of a specific fluorometric proteasomal substrate LLVY-AMC (chymotrypsin-like activity of the 20S proteasomal core) in a fluorometric 96-well plate assay. The reaction mixture containing 50 mM Tris, 5 mM MgCl_2_, 1 mM EDTA, 1 mM DTT, 2 mM ATP and 100 µg of the sperm proteasome extract were prepared in Eppendorf tubes and then mixed with 100 µM LLVY-AMC (Biomol, www.enzolifescience.com). After vortexing, 200 µL of the mixture were quickly transferred to a 96-well plate and reaction product was measured in Fluoroskan Ascent plate reader (Thermo Fisher Scientific Inc., www.thermo.com) at 380/460 nm wavelength, at 37°C. The reaction progressed linearly for 2 hours, pattern typical of the said 20S core activity. (**b**) Effect of ATP, which is necessary for proteasome sustenance, and proteasomal and non-proteasomal protease-inhibitors on LLVY-AMC substrate digestion by the sperm proteasome. The reaction mixtures were prepared as described above. Inhibitors were added to the reaction mixtures before adding the substrate. Final concentrations of the inhibitors were 10 µM MG132 (MG; proteasomal inhibitor), 10 µM Epoxomicin (Epox; proteasomal inhibitor), 10 µg/ml Soybean trypsin inhibitor (STI), 1 mM benzamidine hydrochloride (BH). LLVY-AMC digestion was inhibited to the control level (Con) by MG132 and epoxomicin, or in the absence of ATP (No ATP), whereas non-proteasomal serine protease inhibitors, STI and BH did not show any effect (compared with the enzyme activity of the control, vehicle treated extract - Ext). (**c**) Western blotting of isolated sperm proteasomes. The extract was electrophoresed in 4-20% Tris-glycine gradient gels (PAGEr, www.lonza.com), transferred and detected with an anti-proteasome antibody (Biomol, PW8195; www.biomol.com) recognizing a conserved motif of subunits α1-7 of the 20S core, by following the standard protocol. The extract showed the anticipated specific reaction at 27–29 kDa, verifying the presence of 20S α-subunits which migrate together on SDS-PAGE.
(TIF)

Figure S3 **Colocalization of ubiquitin (UBB: green in merged images) with ZPC protein (ZPC; red in merged images) in growing porcine oocytes and cumulus cells.** Oocytes were isolated from small/growing antral follicles (**a–c**), and from preantral follicles (**d**). Cumulus/corona radiata cells (**e, f**) are shown on the zona surface of oocytes isolated from small antral follicles. (**g**) Accumulation of ZPC in the cortex of an oocyte isolated from an athretic follicle. DNA was counterstained with DAPI (blue in merged images).
(TIF)

Figure S4 **Proteomic identification of sperm acrosomal proteins that are protected from degradation by proteasomal inhibitors added to coincubated sperm-ZPP fractions.** Sperm-ZPP mixtures were separated on SDS-PAGE and treatment-specific bands, as identified in Fig. 6, were excised, digested with trypsin and subjected to LC-MS/MS using an LTQ mass spectrometer (Thermo-Finnigan). Peptides were identified using the Sequest search algorithm. Peptides with probability scores (p<0.05) are shown in red. Note that a full length ACRBP-precursor protein sequence is shown for ACRBP. After translation/during capacitation, the 537 amino acid/∼56 kDa precursor protein is processed proteolytically into 32 kDa functional protein and a small fragment [59]. The processed 32 kDa ACRBP has been shown to be further proteolyzed into two distinct fragments of 17 and 12.5 kDa [59]. N-terminal and C-terminal sequence coverage, shown here, suggests that the low mass doublet band of ACRBP seen in Fig. 6 is a mixture of at least two different fragments derived from the full length, 56 kDa ACRBP-precursor.
(TIF)
