# Supplementary figures and images for: Sperm Proteasomes Degrade Sperm Receptor on the Egg Zona Pellucida during Mammalian Fertilization
Source: PLoS One. 2011 Feb 23;6(2):e17256. doi: 10.1371/journal.pone.0017256 (PMC3044170; doi:10.1371/journal.pone.0017256)

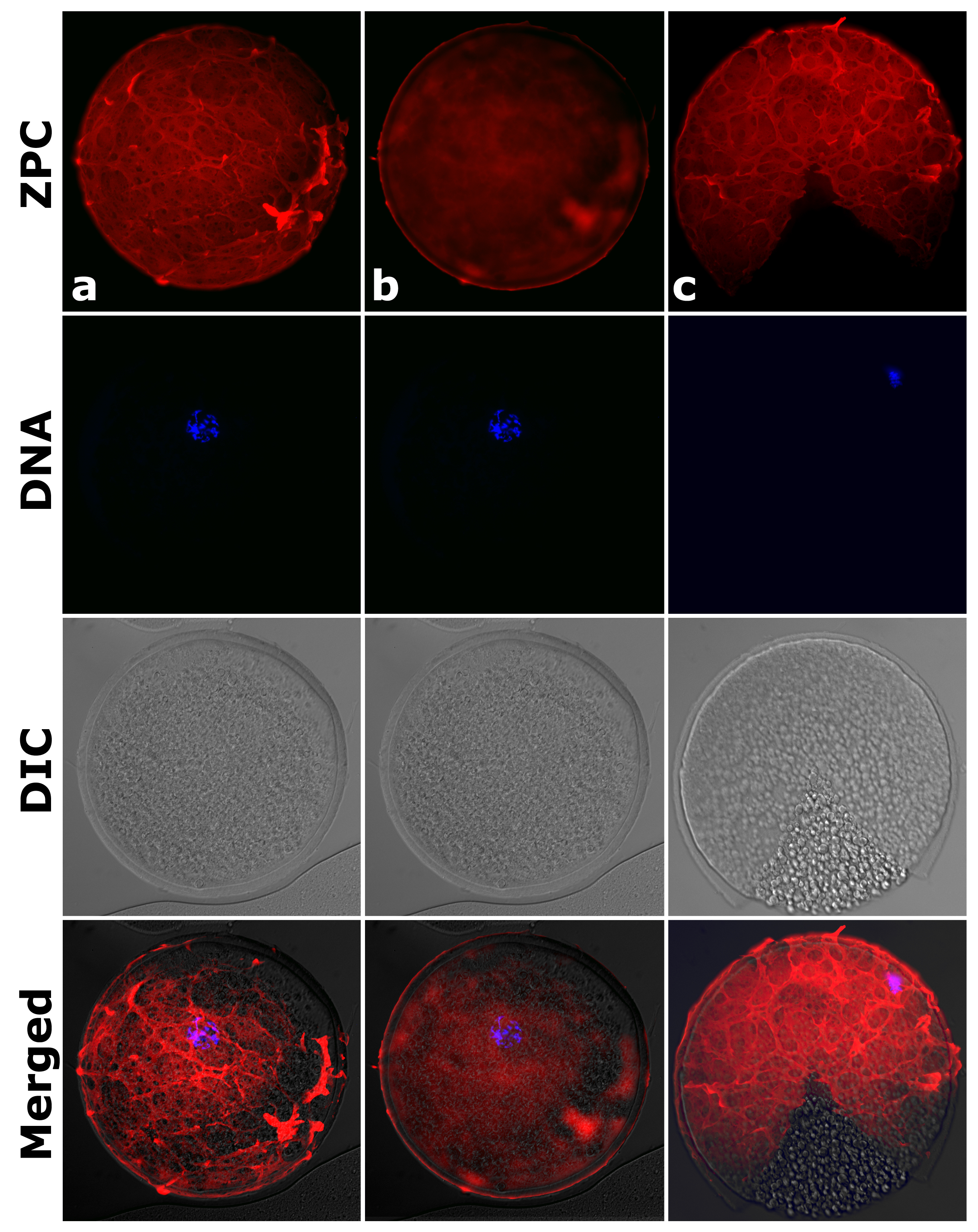

Supplement: Figure S1 — Immunolabeling of porcine ZP with anti-ZPC antibody MA-467, as also used for Western blotting. A representative, mature, metaphase II-stage oocyte is shown on an optical cross section across the equatorial plane (a) and surface plane (b). An oocyte with a cracked ZP is also shown (c) to demonstrate the difference between the fluorescence intensity of the ZP and the ooplasm, exposed by the crack. (TIF) [file pone.0017256.s001.tif]

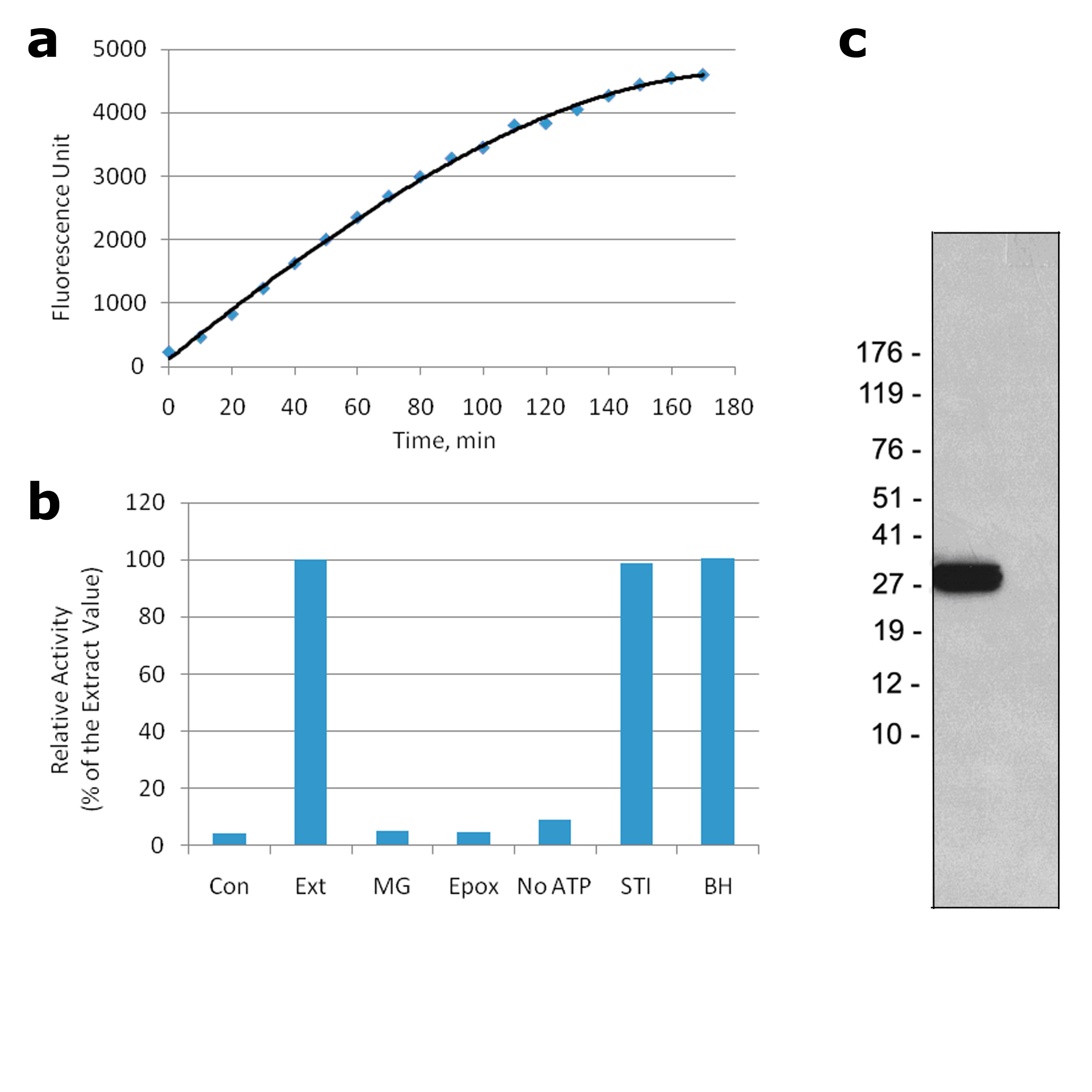

Supplement: Figure S2 — Isolation and characterization of the sperm acrosomal proteasomes. (a) Proteasomal activity of the purified sperm acrosomal proteasomes is demonstrated by time-dependent digestion of a specific fluorometric proteasomal substrate LLVY-AMC (chymotrypsin-like activity of the 20S proteasomal core) in a fluorometric 96-well plate assay. The reaction mixture containing 50 mM Tris, 5 mM MgCl2, 1 mM EDTA, 1 mM DTT, 2 mM ATP and 100 µg of the sperm proteasome extract were prepared in Eppendorf tubes and then mixed with 100 µM LLVY-AMC (Biomol, www.enzolifescience.com). After vortexing, 200 µL of the mixture were quickly transferred to a 96-well plate and reaction product was measured in Fluoroskan Ascent plate reader (Thermo Fisher Scientific Inc., www.thermo.com) at 380/460 nm wavelength, at 37°C. The reaction progressed linearly for 2 hours, pattern typical of the said 20S core activity. (b) Effect of ATP, which is necessary for proteasome sustenance, and proteasomal and non-proteasomal protease-inhibitors on LLVY-AMC substrate digestion by the sperm proteasome. The reaction mixtures were prepared as described above. Inhibitors were added to the reaction mixtures before adding the substrate. Final concentrations of the inhibitors were 10 µM MG132 (MG; proteasomal inhibitor), 10 µM Epoxomicin (Epox; proteasomal inhibitor), 10 µg/ml Soybean trypsin inhibitor (STI), 1 mM benzamidine hydrochloride (BH). LLVY-AMC digestion was inhibited to the control level (Con) by MG132 and epoxomicin, or in the absence of ATP (No ATP), whereas non-proteasomal serine protease inhibitors, STI and BH did not show any effect (compared with the enzyme activity of the control, vehicle treated extract - Ext). (c) Western blotting of isolated sperm proteasomes. The extract was electrophoresed in 4-20% Tris-glycine gradient gels (PAGEr, www.lonza.com), transferred and detected with an anti-proteasome antibody (Biomol, PW8195; www.biomol.com) recognizing a conserved motif of subunits α1-7 of the 20S c [file pone.0017256.s002.tif]

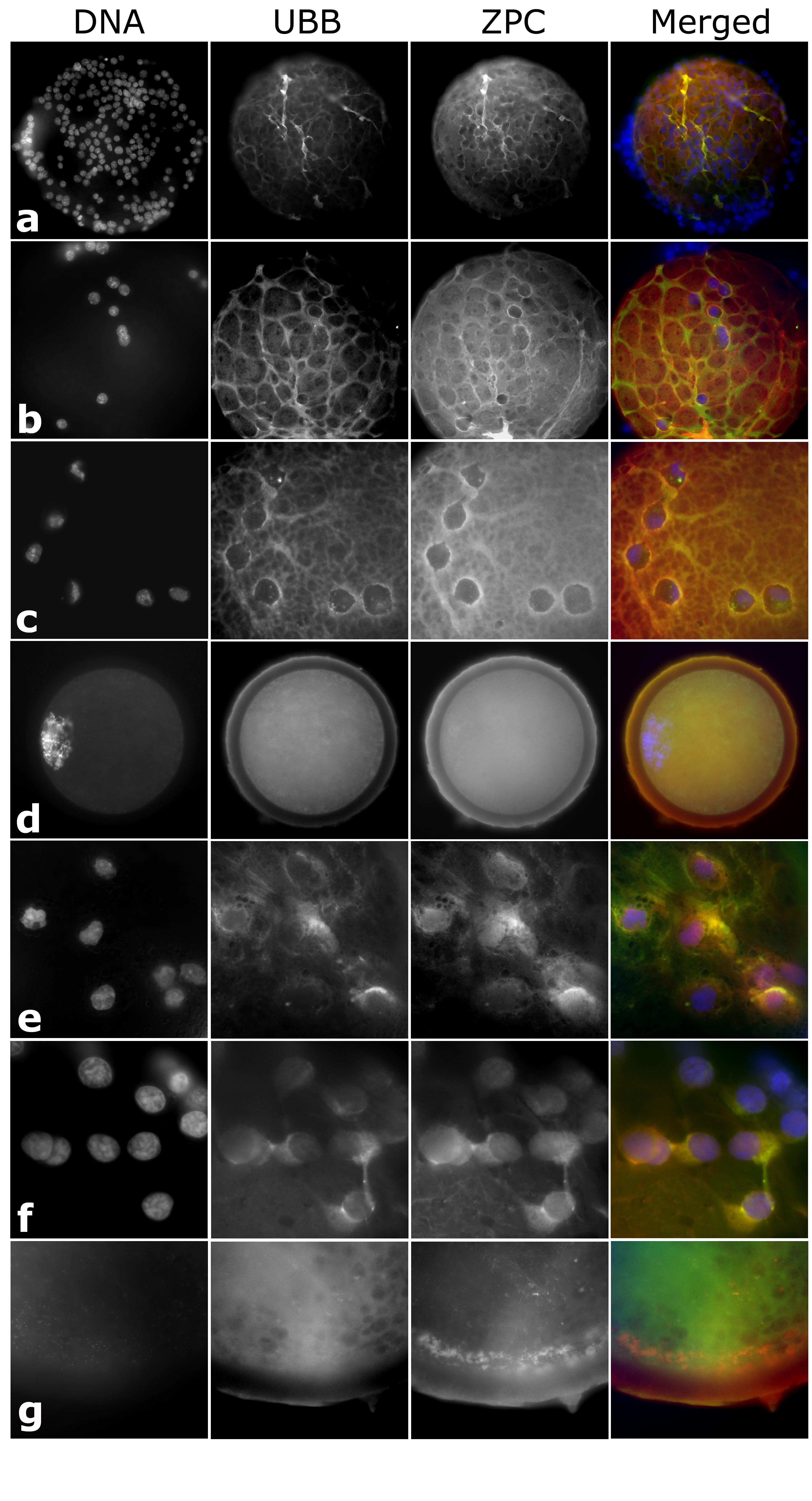

Supplement: Figure S3 — Colocalization of ubiquitin (UBB: green in merged images) with ZPC protein (ZPC; red in merged images) in growing porcine oocytes and cumulus cells. Oocytes were isolated from small/growing antral follicles (a–c), and from preantral follicles (d). Cumulus/corona radiata cells (e, f) are shown on the zona surface of oocytes isolated from small antral follicles. (g) Accumulation of ZPC in the cortex of an oocyte isolated from an athretic follicle. DNA was counterstained with DAPI (blue in merged images). (TIF) [file pone.0017256.s003.tif]

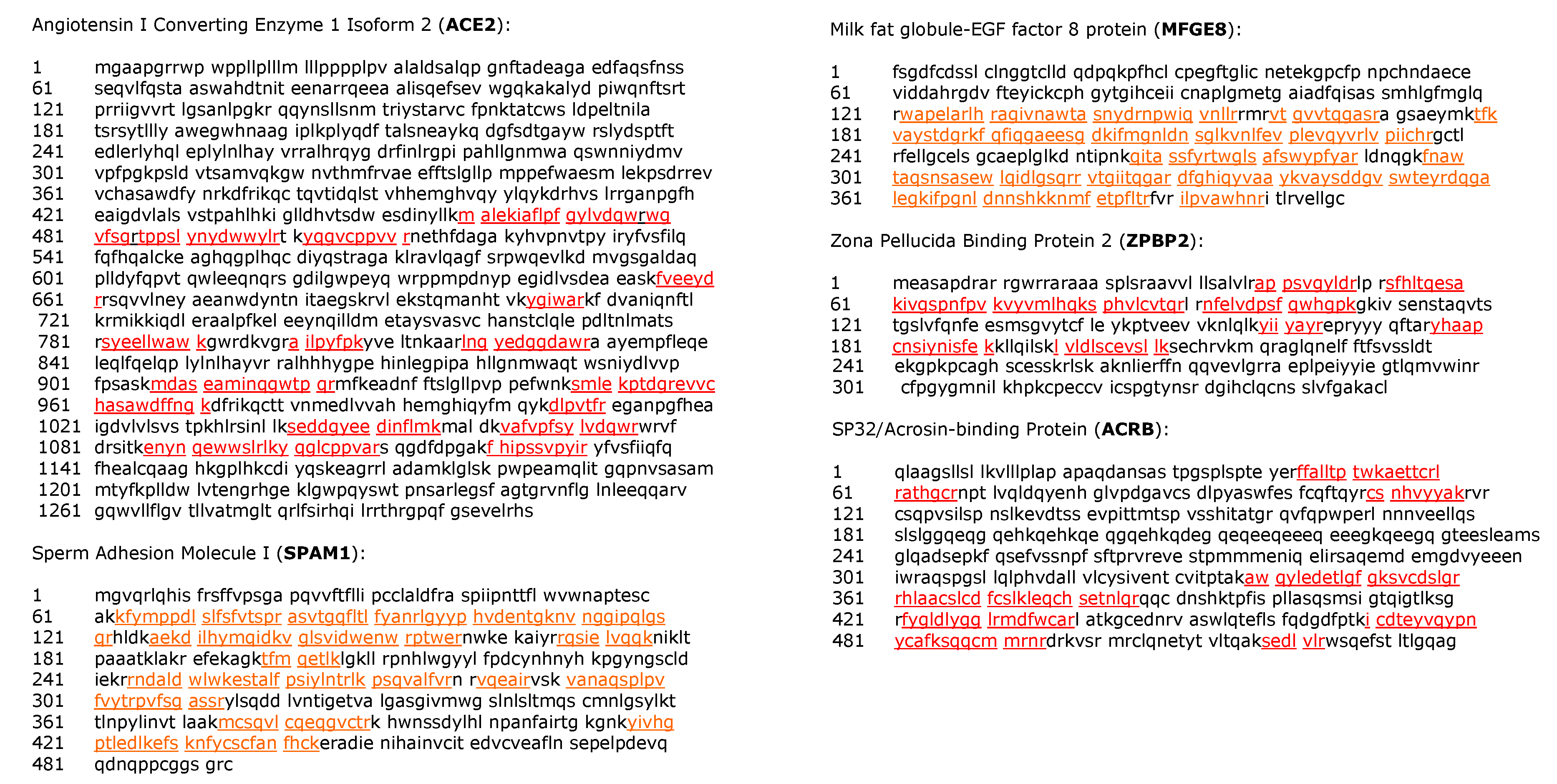

Supplement: Figure S4 — Proteomic identification of sperm acrosomal proteins that are protected from degradation by proteasomal inhibitors added to coincubated sperm-ZPP fractions. Sperm-ZPP mixtures were separated on SDS-PAGE and treatment-specific bands, as identified in Fig. 6, were excised, digested with trypsin and subjected to LC-MS/MS using an LTQ mass spectrometer (Thermo-Finnigan). Peptides were identified using the Sequest search algorithm. Peptides with probability scores (p<0.05) are shown in red. Note that a full length ACRBP-precursor protein sequence is shown for ACRBP. After translation/during capacitation, the 537 amino acid/∼56 kDa precursor protein is processed proteolytically into 32 kDa functional protein and a small fragment [59]. The processed 32 kDa ACRBP has been shown to be further proteolyzed into two distinct fragments of 17 and 12.5 kDa [59]. N-terminal and C-terminal sequence coverage, shown here, suggests that the low mass doublet band of ACRBP seen in Fig. 6 is a mixture of at least two different fragments derived from the full length, 56 kDa ACRBP-precursor. (TIF) [file pone.0017256.s004.tif]
